# Evidence of perturbations of cell cycle and DNA repair pathways as a consequence of human and murine *NF1*-haploinsufficiency

**DOI:** 10.1186/1471-2164-11-194

**Published:** 2010-03-22

**Authors:** Alexander Pemov, Caroline Park, Karlyne M Reilly, Douglas R Stewart

**Affiliations:** 1 Genetic Disease Research Branch, National Human Genome Research Institute, National Institutes of Health, Bethesda, Maryland, 20892, USA; 2 Albert Einstein College of Medicine, Bronx, New York, 10461, USA; 3 National Cancer Institute at Frederick, Fort Detrick Building 50, Frederick, Maryland, 21702, USA

## Abstract

**Background:**

Neurofibromatosis type 1 (NF1) is a common monogenic tumor-predisposition disorder that arises secondary to mutations in the tumor suppressor gene *NF1*. Haploinsufficiency of *NF1 *fosters a permissive tumorigenic environment through changes in signalling between cells, however the intracellular mechanisms for this tumor-promoting effect are less clear. Most primary human *NF1*^+/- ^cells are a challenge to obtain, however lymphoblastoid cell lines (LCLs) have been collected from large NF1 kindreds. We hypothesized that the genetic effects of *NF1*-haploinsufficiency may be discerned by comparison of genome-wide transcriptional profiling in somatic, non-tumor cells (LCLs) from NF1-affected and -unaffected individuals. As a cross-species filter for heterogeneity, we compared the results from two human kindreds to whole-genome transcriptional profiling in spleen-derived B lymphocytes from age- and gender-matched *Nf1*^+/- ^and wild-type mice, and used gene set enrichment analysis (GSEA), Onto-Express, Pathway-Express and MetaCore tools to identify genes perturbed in *NF1*-haploinsufficiency.

**Results:**

We observed moderate expression of *NF1 *in human LCLs and of *Nf1 *in CD19+ mouse B lymphocytes. Using the *t *test to evaluate individual transcripts, we observed modest expression differences in the transcriptome in *NF1*-haploinsufficient LCLs and *Nf1*-haploinsuffiicient mouse B lymphocytes. However, GSEA, Onto-Express, Pathway-Express and MetaCore analyses identified genes that control cell cycle, DNA replication and repair, transcription and translation, and immune response as the most perturbed in *NF1*-haploinsufficient conditions in both human and mouse.

**Conclusions:**

Haploinsufficiency arises when loss of one allele of a gene is sufficient to give rise to disease. Haploinsufficiency has traditionally been viewed as a passive state. Our observations of perturbed, up-regulated cell cycle and DNA repair pathways may functionally contribute to *NF1*-haploinsufficiency as an "active state" that ultimately promotes the loss of the wild-type allele.

## Background

Neurofibromatosis type 1 (NF1) is a common monogenic tumor-predisposition disorder with an autosomal dominant pattern of inheritance that arises secondary to haploinsufficiency of the tumor suppressor gene *NF1*. Haploinsufficiency is often defined as a gene-dosage effect in which loss of one allele of a gene results in disease. In the Knudson "two-hit" model [1], haploinsufficiency of a tumor-suppressor gene (*NF1 *included) increases the probability of cancer development, but does not functionally contribute to it [2]. This view of haploinsufficiency as a passive state is coming under revision [3]. In mice haploinsufficiency of *NF1 *fosters a permissive tumorigenic environment for the development of neurofibromas [4,5], optic nerve gliomas [6] and blood vessels [7], suggesting a role for haploinsufficiency itself in tumor formation. The intracellular changes that give rise to this tumor-promoting effect of *NF1*-haploinsufficiency are unclear.

In this paper, we used a novel approach to investigate the genetic consequences of *NF1*-haploinsufficiency in humans. We hypothesized that the genetic effects of *NF1*-haploinsufficiency may be discerned by comparison of genome-wide transcriptional profiling in somatic, non-tumor cells from NF1-affected and -unaffected individuals. Studies of haploinsufficiency in humans are easily confounded by inter-individual variation [3] due to germline expression differences and mutation heterogeneity. To reduce variation in germline expression, we used a family-based approach and performed whole-genome transcriptional profiling of lymphoblastoid cell lines (LCLs) on gender- and age- matched affected and unaffected (but biologically related) individuals from two large NF1 pedigrees. Neurofibromin (the protein product of *NF1*) is expressed and is functionally important in B lymphocytes (the cells transformed by Epstein-Barr virus (EBV) into LCLs) [8]. The use of peripheral blood mononuclear cells or LCLs for disease expression profiling has precedents in oncologic [9], neurologic [10,11], and monogenic [12,13] disorders, as well as in NF1 itself [14]. To reduce genetic heterogeneity, we studied affected individuals within a pedigree who share the same mutation in *NF1*. To further control for heterogeneity, we compared the results from the two human pedigrees to whole-genome transcriptional profiling in spleen-derived B lymphocytes from age- and gender-matched *Nf1*^+/- ^[15] and wild-type mice as a cross-species filter for changes specific to *NF1*-haploinsufficiency. We used a variety of bioinformatic and molecular methods to identify and validate genes perturbed in *NF1*-haploinsufficiency.

## Results

### *NF1 *mutations in Coriell pedigree 2176 and ECACC pedigree P0117

To minimize the effects of genetic background and eliminate *NF1 *mutation heterogeneity, we investigated the consequences of *NF1 *haploinsufficiency on expression within age- and gender-matched groups from two pedigrees (Table 1). All individuals deemed "NF1-affected" in Coriell pedigree 2176 harbored a 4-bp deletion in exon 22, which is predicted to result in a premature stop codon. All individuals deemed "NF1-affected" from ECACC family P0117 harbored a 9-bp deletion in intron 9, which is predicted to disrupt the acceptor splice site of exon 10a, leading to exon skipping during mRNA splicing. All individuals deemed "NF1-unaffected" in both the Coriell and ECACC kindreds did not harbor their pedigree-specific *NF1 *mutation.

**Table 1:** NF1 pedigrees used in the study.

Sample	Sex	Age	NF1-status	Set	Family ID	Sample ID	Mutation
**1-A**	**F**	**47**	**Affected**	**Coriell-18**	**2176**	**GM09534**	**c.3739_3742del**
**3-A**	**F**	**55**	**Affected**	**Coriell-18**	**2176**	**GM09616**	**c.3739_3742del**
**5-A**	**M**	**26**	**Affected**	**Coriell-18**	**2176**	**GM09619**	**c.3739_3742del**
**8-A**	**F**	**52**	**Affected**	**Coriell-18**	**2176**	**GM09627**	**c.3739_3742del**
**9-A**	**M**	**41**	**Affected**	**Coriell-18**	**2176**	**GM09628**	**c.3739_3742del**
**13-A**	**F**	**16**	**Affected**	**Coriell-18**	**2176**	**GM09633**	**c.3739_3742del**
**14-A**	**M**	**15**	**Affected**	**Coriell-18**	**2176**	**GM09634**	**c.3739_3742del**
**23-A**	**M**	**N.A**.	**Affected**	**Coriell-18**	**2176**	**GM09692**	**c.3739_3742del**
**24-A**	**M**	**51**	**Affected**	**Coriell-18**	**2176**	**GM09693**	**c.3739_3742del**
4-U	F	28	Unaffected	Coriell-18	2176	GM09617	N.D.
6-U	M	67	Unaffected	Coriell-18	2176	GM09625	N.D.
10-U	M	32	Unaffected	Coriell-18	2176	GM09630	N.D.
12-U	F	33	Unaffected	Coriell-18	2176	GM09632	N.D.
15-U	M	35	Unaffected	Coriell-18	2176	GM09635	N.D.
16-U	F	29	Unaffected	Coriell-18	2176	GM09638	N.D.
19-U	M	38	Unaffected	Coriell-18	2176	GM09651	N.D.
20-U	F	39	Unaffected	Coriell-18	2176	GM09652	N.D.
21-U	M	N.A.	Unaffected	Coriell-18	2176	GM09688	N.D.
**E1-A**	**M**	**37**	**Affected**	**ECACC-6**	**P0117**	**89082417**	**c.1261-2_1261-10del**
**E6-A**	**M**	**33**	**Affected**	**ECACC-6**	**P0117**	**89082422**	**c.1261-2_1261-10del**
**E10-A**	**F**	**40**	**Affected**	**ECACC-6**	**P0117**	**89082426**	**c.1261-2_1261-10del**
E2-U	F	34	Unaffected	ECACC-6	P0117	89082418	N.D.
E7-U	M	41	Unaffected	ECACC-6	P0117	89082423	N.D.
E11-U	M	31	Unaffected	ECACC-6	P0117	89082427	N.D.

EBV-transformed lymphoblastoid cell lines (LCL) from two kindreds, Coriell-18 and ECACC-6, were obtained from Coriell and ECACC cell repositories, respectively. NF1-affected members of Coriell-18 set have a 4 bp deletion (5'-TTTG-3') in exon 22 of *NF1*, which is predicted to result in a premature stop codon. NF1-affected members of ECACC-6 set have a 9 bp deletion (5'-TTTTCTCTA-3') in intron 9 of *NF1*, which is predicted to disrupt the acceptor splice site of exon 10a, leading to exon skipping during mRNA splicing. Both mutations are predicted to be pathogenic. Mutations are described with nucleotide 1 being the A of the ATG translation initiation codon in the sequence NM_000267. Coriell and ECACC Family and Sample IDs for the cell lines are indicated in respective columns. Information for NF1-affected individuals is distinguished in boldface. N.A. - data not available; N.D. - not detected.

### Evaluation of *Nf1*^+/- ^mice

The six adult *Nf1*^+/- ^heterozygous mice were confirmed to bear the deletion of exon 31 and a part of intron 30 in *Nf1 *(data not shown). We detected no significant differences in organ weights between heterozygote and wild-type animals (all 8 months old), with the exception of spleen weight (wild-type mice: 72 mg vs. *Nf1*^+/- ^mice:106 mg; *P *< 0.00096), which was larger in the *Nf1*^+/-^mice. There was no histologic evidence of a neoplastic process in any of the tissues examined in the animals. The complete blood count of both the wild-type and *Nf1*^+/- ^mice was normal.

### *NF1 *is moderately expressed in human LCLs and CD19+ mouse B lymphocytes

**Figure 1:**
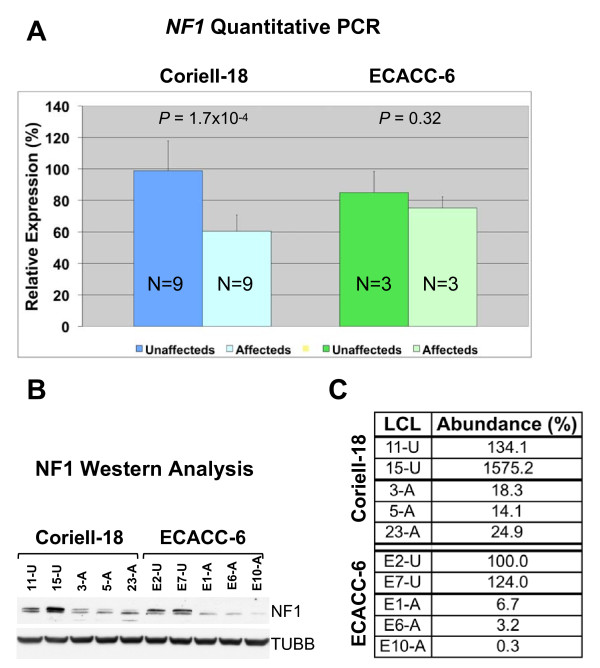
**Quantitative PCR and western blot of the relative abundance of neurofibromin in lymphoblastoid cell lines**. A) Relative abundance of *NF1 *mRNA in Coriell-18 and ECACC-6 sets as detected by quantitative PCR. *P *values of respective *t *tests are shown above the bar plots. B) Western blot of neurofibromin in select lymphoblastoid cell lines (LCLs). TUBB - β-tubulin. C) Relative abundance of neurofibromin in select LCLs. *NF1 *abundance in sample E2-U was arbitrary set at 100%; NF1 abundance in the remaining LCLs is expressed as percentage of that in E2-U.

**Figure 2:**
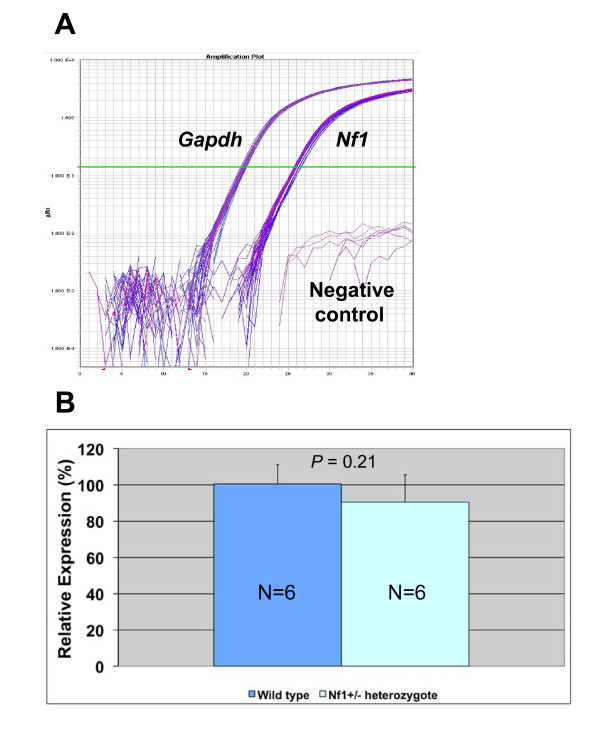
**Quantitative PCR analysis of relative abundance of *Nf1 *mRNA in *Nf1*^+/- ^mice**. A) Plot of real-time amplification of *Nf1 *and *Gapdh *mRNAs in six wild-type and six *Nf1*^+/- ^mice. Samples containing no input RNA are shown as "Negative control". B) Relative abundance of *Nf1 *mRNA from wild-type and *Nf1*^+/- ^mice. *P *value of the *t *test is shown above the bar plot.

In NF1-affected LCLs, we observed a mean decrease of 40% (Coriell-18) and 17% (ECACC-6) of *NF1 *mRNA abundance compared to NF1-unaffected LCLs from the same pedigree (Figure 1). In the Coriell-18 group, the difference in *NF1 *expression was statistically significant. In the ECACC-6 group, there was a non-significant decrease in *NF1 *expression (Figure 1A). Nevertheless, there was a dramatic decrease in neurofibromin expression detected by western blotting between selected affected and unaffected individuals in both the Coriell and ECACC pedigrees (Figures 1B and 1C). Since mouse B lymphocytes cannot be immortalized with viral transfection, we used freshly isolated murine spleen B lymphocytes. Fluorescent-activated cell sorting (FACS) analysis showed greater than 90% purity of CD19+ cells from all animals (data not shown). As in human LCLs, *Nf1 *is moderately expressed in mouse lymphocytes (Figure 2A) but is not significantly different between *Nf1*^+/- ^and wild-type animals (Figure 2B), as previously reported [15]. We were unable to perform western blotting on murine spleen CD19+ B lymphocytes due to the limited amount of material available, nevertheless, neurofibromin abundance in other tissues in the *Nf1*^+/- ^mice was decreased (data not shown).

### *NF1*-haploinsufficiency in LCLs and mouse B lymphocytes results in modest differences in individual transcript abundance between NF1-affected and -unaffected

#### Permuted t test and intersection analysis

**Figure 3:**
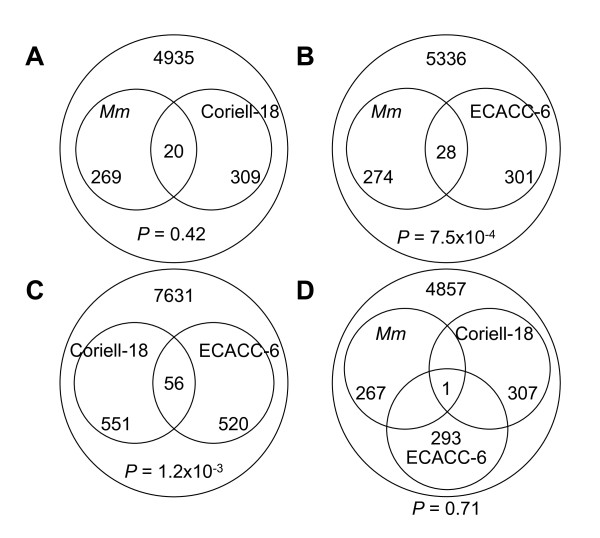
**Intersection analysis between top-ranking transcripts in human and mouse sets**. Top ~5% of all transcripts from analysis of three sample sets on the Illumina platform were chosen for the intersection analysis. A) Coriell-18 and *Nf1*-Mouse-12 comparison; B) ECACC-6 and *Nf1*-Mouse-12 comparison; C) Coriell-18 and ECACC-6 comparison; D) Three-way comparison. "*Mm" *designates *Nf1-*Mouse-12 set.

In the human and mouse expression datasets, permuted *t *test revealed few statistically significant genes after correction for multiple testing (Table 2). There were no transcripts with a false discovery rate (FDR) less than 0.25 in common among the three groups. Thus, we performed an intersection analysis of the top ~5% of transcripts from the three groups (Figure 3; Additional file 1). The overlap of ECACC-6 with *Nf1*-Mouse-12 was highly significant (*P *= 7.5 × 10^-4^) as well as the overlap between two human groups (*P *= 1.2 × 10^-3^). Coriell-18 did not significantly overlap with *Nf1*-Mouse-12 (*P *= 0.42). The three-way overlap between the groups produced a single gene and was not significant (*P *= 0.71).

**Table 2:** Top-ranked differentially expressed genes in Coriell-18, ECACC-6 and *Nf1*-Mouse-12 sample sets.

Coriell-18	*P *value	FDR	FD	Gene
**1**	**0.0001**	**0.162**	**0.57**	***IFI6***
**2**	**0.0001**	**0.162**	**0.78**	***CNKSR3***
**3**	**0.0001**	**0.162**	**1.23**	***AKAP2***
**4**	**0.0001**	**0.162**	**0.67**	***OAS1***
**5**	**0.0001**	**0.162**	**1.27**	***ZRANB1***
**6**	**0.0001**	**0.162**	**0.66**	***TCN2***
**7**	**0.0001**	**0.164**	**0.72**	***GAS2***
**8**	**0.0002**	**0.164**	**0.64**	***OAS3***
**9**	**0.0002**	**0.164**	**0.66**	***HERC5***
**10**	**0.0002**	**0.164**	**0.69**	***EPSTI1***
**11**	**0.0002**	**0.164**	**0.81**	***NDUFC1***
**12**	**0.0002**	**0.164**	**0.80**	***ACOT9***
**13**	**0.0002**	**0.164**	**0.74**	***RFC5***
**14**	**0.0003**	**0.164**	**0.86**	***NMI***
**15**	**0.0003**	**0.170**	**1.26**	***DYRK2***
**16**	**0.0003**	**0.193**	**0.75**	***LGP2***
17	0.0006	0.272	1.12	*LRRC37B*
18	0.0006	0.272	0.80	*PPP2R4*

**ECACC-6**	***P *value**	**FDR**	**FD**	**Gene**

**1**	**0.8 × 10-6**	**0.008**	**2.65**	***HMHB1***
**2**	**0.8 × 10-5**	**0.038**	**2.96**	***HAVCR2***
**3**	**0.2 × 10-4**	**0.067**	**1.80**	***CHURC1***
**4**	**0.0001**	**0.232**	**1.76**	***GIMAP5***
5	0.0003	0.473	1.69	*GIMAP6*
6	0.0003	0.473	1.34	*RORA*
7	0.0004	0.473	0.58	*ADM*
8	0.0004	0.473	0.66	*DNAJC15*
9	0.0005	0.473	0.54	*C3orf59*
10	0.0005	0.473	1.45	*RAB31*
11	0.0006	0.553	1.65	*PSTPIP2*
12	0.0007	0.553	1.33	*PACSIN2*
13	0.0008	0.554	1.35	*MYB*
14	0.0009	0.554	0.75	*ISCU*
15	0.0009	0.554	1.36	*PKHD1L1*
16	0.0009	0.554	0.69	*ASB2*
17	0.0010	0.575	0.74	*DUSP14*
18	0.0011	0.575	0.75	*IMPA1*

***Nf1*-Mouse-12**	***P *value**	**FDR**	**FD**	**Gene**

**1**	**0.1 × 10-6**	**0.001**	**1.42**	***2010305C02Rik***
**2**	**0.2 × 10-5**	**0.011**	**1.29**	***C730049P21***
**3**	**0.3 × 10-5**	**0.014**	**1.30**	***Inpp5k***
4	0.0003	0.879	1.23	*H2-T10*
5	0.0004	0.879	1.29	*Pnkd*
6	0.0004	0.879	1.42	*Cxcr3*
7	0.0005	0.903	1.24	*Gsg2*
8	0.0009	1.000	1.27	*Gstt3*
9	0.0009	1.000	1.38	*9130213B05Rik*
10	0.0009	1.000	1.19	*Igtp*
11	0.0010	1.000	1.18	*Rnf43*
12	0.0010	1.000	1.50	*Serpina3f*
13	0.0011	1.000	1.17	*Ncapd2*
14	0.0013	1.000	1.19	*Inpp5k*
15	0.0014	1.000	1.17	*Slc25a19*
16	0.0015	1.000	1.40	*LOC238447*
17	0.0020	1.000	0.87	*Gosr1*
18	0.0021	1.000	1.24	*LOC327957*

To evaluate statistical significance of the differential expression of genes, *t *tests were performed for two human and the mouse sample sets. In each list, genes were ranked according to their False Discovery Rate (FDR). Significant genes with FDR< 0.25 are shown in boldface. "FD" - Fold Difference of expression values of NF1-affecteds vs. NF1-unaffecteds, or *Nf1*^+/- ^vs. wild-type mice.

#### Cross-platform validation of microarray results

**Figure 4:**
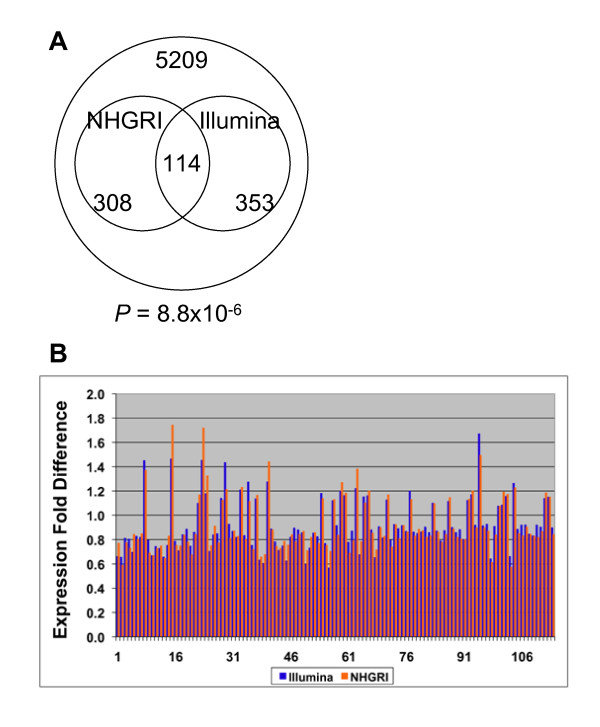
**Comparison of expression profiles generated by Illumina_Human_WG_v2 and spotted oligonucleotide microarrays**. A) Coriell-18 samples were analyzed in parallel on two different types of microarrays: Illumina_Human_WG_v2 ("Illumina") and spotted oligonucleotide microarrays manufactured in the NHGRI core facility ("NHGRI"). Genes were ranked according to nominal *P *values of the *t *tests, and top ~5% of genes in each list were chosen for the intersection analysis; B) Bar plot represents fold difference of expression values (NF1-affecteds vs. NF1-unaffecteds) for the overlapping genes. Each overlapping transcript is represented by a pair of bars. Blue bars denote transcripts on Illumina platform; orange bars denote transcripts on the NHGRI platform. Numbers on X-axis represent transcripts in the overlap (114 total).

We analyzed the Coriell-18 group on both the Illumina single-color and the spotted oligonucleotide (two-color) arrays. Of the top 5% of differentially expressed genes, there were 114 common genes on both lists (Additional file 2); the overlap is highly statistically significant (*P *= 8.8 × 10^-6^) (Figure 4A). Moreover, for those 114 transcripts, we observed 100% concordance between the two platforms in direction of expression (over or under). The degree of change (fold difference) for the transcripts was close in both platforms as well (Figure 4B).

#### Quantitative PCR validation of microarray results

The validation set for quantitative PCR (qPCR) included transcripts which were either statistically significant by *t *test or in an intersection analysis (Table 3). Of twelve *t *test-significant transcripts (seven human and five mouse), nine transcripts (five human and four mouse), or 75%, were confirmed by qPCR (nominal *P *< 0.05). The validated transcripts included human *CNKSR3*, *IFI6, EGLN3, MGST3, POMC *and mouse *Cxcr3, Gsg2, Igtp, and Rnf43*. Of nine transcripts identified by intersection analysis, 33% were validated by qPCR (three human transcripts only:*C11orf75, RAB31, and DUSP4*). *RAB31 *was the only transcript confirmed by qPCR in both human groups. *DUSP4*, found in three-way overlap, was validated by qPCR in the Coriell-18 group only. Differential expression as determined by qPCR was modest and ranged from 0.39 for *CNKSR3 *in Coriell-18 to 2.57 for *RAB31 *in ECACC-6. We observed near 100% concordance between microarray and qPCR approaches in direction of gene expression (over or under). The only exceptions were two genes in ECACC-6: *CHURC1 *was over-expressed by microarray, but under-expressed by qPCR, and *POMC *was under-expressed by microarray, and over-expressed by qPCR (Additional file 3). Differential expression of transcripts tended to be more pronounced in qPCR than microarrays (Additional file 3).

**Table 3:** Quantitative PCR validation of microarray data for select human and mouse genes.

Gene	Reason for Validation	Quantitative PCR *P *values		
		
		Coriell-18	ECACC-6	*Nf1*-Mouse-12
***AUTS2***	**Overlap of *Hs *sets**	**0.0630**	**0.1790**	**N/A**
***C11orf75***	**Overlap of *Hs *sets**	**0.0127**	0.2270	N/A
***RAB31***	**Overlap of *Hs *sets**	**0.0168**	**0.0019**	N/A
***DUSP4***	**3-Way Overlap of *Hs *and *Mm *sets**	**0.0398**	0.1910	N/A
*AKAP2*	Significant in Coriell-18 (I)	0.1170	0.3540	N/A
***CNKSR3***	**Significant in Coriell-18 (I)**	**0.0026**	0.1850	N/A
***IFI6***	**Significant in Coriell-18 (I)**	**0.0013**	0.5930	N/A
*CHURC1*	Significant in ECACC-6	0.7390	0.1450	N/A
*GSG2*	Significant in *Nf1-Mm*-12*	0.7210	0.1360	N/A
***EGLN3***	**Significant in Coriell-18 (N)**	**0.0076**	0.7630	N/A
***MGST3***	**Significant in Coriell-18 (N)**	**0.0005**	0.6991	N/A
***POMC***	**Significant in Coriell-18 (N)**	**0.0170**	0.5582	N/A
*Aytl2*	Overlap of *Hs-Mm *sets	N/A	N/A	0.1900
*Crip1*	Overlap of *Hs-Mm *sets	N/A	N/A	0.5830
*Pecr*	Overlap of *Hs-Mm *sets	N/A	N/A	0.5840
*Zfp36l1*	Overlap of *Hs-Mm *sets	N/A	N/A	0.5140
*Dusp4*	3-Way Overlap of *Hs *and *Mm *sets	N/A	N/A	0.7410
***Cxcr3***	Significant in *Nf1-Mm*-12*	N/A	N/A	**0.0052**
***Gsg2***	Significant in *Nf1-Mm*-12*	N/A	N/A	**0.0262**
***Igtp***	Significant in *Nf1-Mm*-12*	N/A	N/A	**0.0380**
*Inpp5k*	Significant in *Nf1-Mm*-12	N/A	N/A	0.0720
***Rnf43***	Significant in *Nf1-Mm*-12*	N/A	N/A	**0.0004**

Statistical significance of differential expression of genes was evaluated by *t *test. Genes with nominal *P *values < 0.05 were considered significant and are shown in boldface. "(I)" and "(N)" in "Significant in Coriell-18 (I)/(N)" indicate that sample set Coriell-18 was analyzed on the Illumina or NHGRI platform, respectively. "*Hs*" designates human (Coriell-18 and ECACC-6) datasets; "*Mm*" designates murine (*Nf1*-Mouse-12) dataset. * - nominal *P *value < 0.001. "N/A" - not applicable.

### GSEA analysis identifies genes that control cell cycle, DNA replication and repair, transcription and translation, and immune response as the most perturbed in *NF1*-haploinsufficient human and mouse

Table 4 lists the number of up- and down-regulated gene sets, as determined by gene set enrichment analysis (GSEA), with a FDR less than 0.05 in the Coriell-18, ECACC-6 and *Nf1*-Mouse-12 groups. Leading edge analysis (LEA) of the common up- and down- regulated gene sets was then performed, followed by an ontological determination of individual LEA genes (Additional file 4, Figure 5). We found that >75% of up-regulated LEA genes in the human ECACC-6 and *Nf1*-Mouse-12 groups (cell cycle/mitosis/cytokinesis and DNA repair/replication/recombination categories) shared similar ontology (Additional file 5). Three of the four largest ontological categories from the analysis of Coriell-18 LEA (transcription/RNA processing, DNA repair/replication/recombination and cell proliferation) were shared with the ECACC-6 and *Nf1*-Mouse-12 groups, albeit the distribution of genes among the ontological categories was different. In the Coriell-18 up-regulated LEA, the largest category (translation/protein biosynthesis/ribosome biogenesis) was unique to that group.

**Figure 5:**
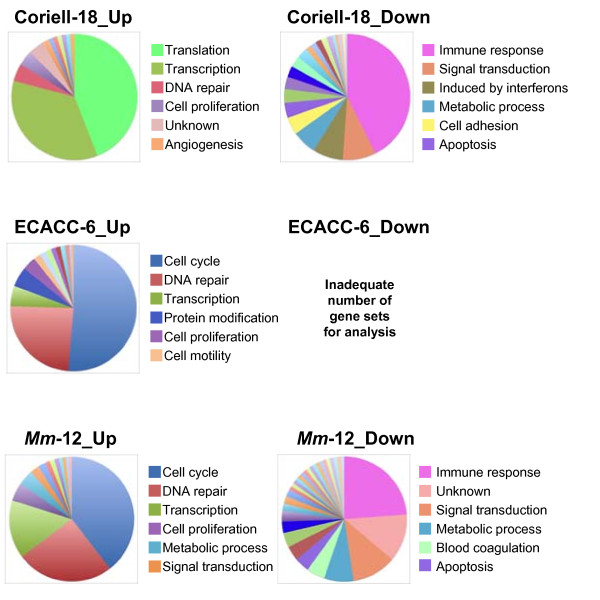
**GSEA analysis of human and mouse expression datasets and ontological categories of LEA gene lists**. Up- and down-regulated statistically significant GSEA gene sets were subjected to LEA analysis and resulting genes were grouped in related ontological categories. The pie chart diagrams of up- and down-regulated LEA genes are shown on the left and on the right, respectively. The same ontological categories are represented by the same color on the pie charts. Only the first six most abundant ontological categories are shown for each set. To save space, the following ontological categories were shortened as follows: Translation -Translation/Protein biosynthesis/Ribosome biogenesis; Transcription - Transcription/RNA processing; DNA repair - DNA repair/replication/recombination; Cell cycle - Cell cycle/Mitosis/Cytokinesis; Immune response - Immune/Defense/Antiviral/Inflammatory response.

**Table 4:** Number of significant GSEA gene sets in Coriell-18, ECACC-6 and *Nf1-*Mouse-12 expression datasets.

	Coriell-18	ECACC-6	*Nf1*-Mouse-12
Up-regulated gene sets	4	65	189
Down-regulated gene sets	40	1	4

Gene sets with FDR < 0.05 were considered significant. "Up-regulated gene sets" and "Down-regulated gene sets" designate GSEA gene sets overexpressed or underexpressed, respectively, in NF1-affected individuals or *Nf1*^+/- ^mice as compared to NF1-unaffecteds or wild-type mice.

Ontological analysis of down-regulated genes in Coriell-18 and *Nf1*-Mouse-12 groups revealed four major common categories (excluding "unknown"): immune/defense/antiviral/inflammatory response, signal transduction, metabolic process and apoptosis (Figure 5; Additional file 5). "Immune/defense/antiviral/inflammatory response" was the largest ontological category in the two groups. These genes accounted for more than a third and almost a quarter of the genes in the Coriell-18 and the mouse LEA lists, respectively. Note that GSEA of down-regulated transcripts in ECACC-6 yielded only one significant gene set, and therefore LEA of down-regulated transcripts in ECACC-6 could not performed. When examined individually, the majority of LEA genes (whether up- or down-regulated) are only modestly differentially expressed in affecteds as compared to unaffecteds, or in *Nf1*^+/- ^compared to wild-type mice.

### Ontological analysis of differentially expressed genes with Onto-Express, Pathway-Express and MetaCore tools

To validate the results of the GSEA analysis of *NF1 *haploinsufficiency, we analyzed the Coriell-18, ECACC-6 and *Nf1*-Mouse-12 sample sets using Onto-Express [16,17], Pathway-Express [18,19] and MetaCore software (Additional files 6, 7, 8, 9). Onto-Express analysis identified the ontological category "transcription" (FDR = 1.86 × 10^-4^) as the most significantly perturbed biological process among up-regulated transcripts in the Coriell-18 kindred (Additional file 6). Among down-regulated genes in the Coriell-18 sample set, Onto-Express analysis identified "immune response," "response to virus," and 'innate immune response" as highly statistically significant ontological categories (Additional file 6). Among up-regulated transcripts in the *Nf1*-Mouse-12 set, Onto-Express identified "mitosis," "cell cycle," "cell division" and "DNA replication" as highly statistically significant perturbed ontological categories (Additional file 6). There were no statistically significant down-regulated biological processes in the ECACC-6 and *Nf1*-Mouse-12 sets as determined by Onto-Express analysis. The Onto-Express method identified four ontological categories with modest FDR among genes up-regulated in ECACC-6 (Additional file 6). In summary, with the exception of ontological categories up-regulated in ECACC-6, there was good overlap in the results from the GSEA and Onto-Express methods (Figure 5, Additional file 5 and Additional file 6)

Next, we used Pathway-Express [18,19] to determine whether differentially expressed genes in the Coriell-18, ECACC-6 and *Nf1*-Mouse-12 sample sets are enriched with genes from known cellular pathways and whether these pathways are significantly perturbed by *NF1*-haploinsufficiency. Pathway-Express is a systems biology approach that considers the magnitude of expression change, gene type, position and interactions in known pathways [18]. As with Onto-Express, the Pathway-Express analysis agrees reasonably well with the GSEA analysis for the Coriell-18 and the *Nf1*-Mouse-12 sample sets. In these two sample sets, both Pathway-Express and GSEA identified immune system function and cell cycle, DNA replication and DNA repair, as significantly perturbed by *NF1*-haploinsufficiency (Additional file 7, Figure 5, Additional file 5). In contrast, there was no overlap in the analysis of ECACC-6 by GSEA and Pathway-Express. Examination of the pathways significantly perturbed in the three sets (as per Pathway-Express analysis) reveals that immune system signalling networks were the most abundant among the sets (Additional file 7).

Lastly, we used ontological categories and pathway analyses of the MetaCore "Biological Processes" and "Pathway Maps" as a third independent validation of our results from GSEA. First, the "Biological Processes" analysis (Additional file 8) yielded many ontological categories previously identified using the GSEA and Onto-Express methods. Among the top ten ontological categories for genes up-regulated in the Coriell-18 set are included "gene expression," "chromatin remodeling," "transcription," and "regulation of DNA damage response signal transduction by p53 class mediator." Similarly, the top ten ontological categories for genes up-regulated in the *Nf1*-Mouse-12 set all pertain to cell cycle and mitosis processes. As with the Onto-Express and Pathway-Express methods, there was more limited overlap in ontological categories for genes up-regulated in the ECACC-6 set. Second, the "Pathway Maps" method (Additional file 9) uses the GeneGo proprietary database to identify statistically significant pathways perturbed in a list of differentially expressed genes. This approach identified the "cell cycle_chromosome condensation in prometaphase" as the top perturbed pathway in the up-regulated *Nf1*-Mouse-12 set. This and other cell cycle and immune pathways are akin to those found using the Pathway-Express (Additional file 7) approach in the *Nf1*-Mouse-12 set. The pathways identified by the "Pathway Maps" method for the Coriell-18 and ECACC-6 sets did not correlate well with those found by Pathway-Express.

The *Nf1*-Mouse-12 set produced the most consistent results across all analyses (GSEA, Onto-Express, Pathway-Express and MetaCore). This may be due to the uniform genetic background shared by the animals in the set. We found less consistent results across all analyses with the ECACC-6 set, however this could be due to its small size and diminished statistical power.

## Discussion and Conclusions

The clinical, cellular and cell-signalling consequences of *NF1*-haploinsufficiency are well known [20-22]. Genomic changes are less well characterized. We present, to our knowledge, the first genome-wide study of the consequences of *NF1*-haploinsufficiency on germline (i.e. non-tumor) gene expression in humans. Consistent with the known dysregulation of RAS in NF1, we found evidence of up-regulation of cell cycle, mitosis, cytokinesis and RNA processing and transcription ontologic categories in LCLs. For the first time, we also found up-regulation of DNA repair, replication and recombination ontologic categories, presumably secondary to generalized cell cycle activation. Activation and subsequent deregulation of these critical pathways is a plausible cause for the permissive tumorigenic environment that is the hallmark of *NF1*-haploinsufficiency.

We hypothesized that in the tightly regulated somatic cell haploinsufficient changes, even of a critical gene like *NF1*, will be modest but detectable by microarray. Studies of the genome-wide consequences of tumor suppressor haploinsufficiency ideally require normal tissue, since tumor cell lines often feature copy-number changes [23]. Other studies investigating the genomic consequences of tumor suppressor inactivation have engineered near-nullizygosity (i.e. not haploinsufficiency) using RNA interference technology [24]. We elected to use LCLs from two banked kindreds for three reasons. First, within a family we were able to control for *NF1 *mutation heterogeneity. Second, neurofibromin is expressed and is functionally important in RAS regulation in B lymphocytes [25,26] in mouse models of NF1. Consistent with a functional role for neurofibromin in control of murine lymphocyte growth regulation in spleen [26], we observed a statistically significant difference in spleen weights between wild-type and *Nf1*^+/- ^animals. Importantly, we detected no evidence by histology or blood count of a leukemic or pre-leukemic disorder. Third, the study of the genetics of gene expression commonly uses LCLs, whose global gene expression patterns do not appear to be significantly disrupted by EBV transformation [27]. We show that *NF1 *is expressed at modest levels in normal LCLs (Figure 1) and fluctuates following serum deprivation (Additional file 10), suggesting that EBV transformation of B lymphocytes did not abrogate baseline or serum-dependent *NF1 *expression.

We found a statistically significant difference in *NF1 *expression level between affected and unaffected individuals in the Coriell 2176 pedigree (Figure 1). Affected individuals from the kindred harbor an *NF1 *mutation predicted to result in a premature stop codon, which presumably results in nonsense-mediated decay. We observed no statistically significant difference in *NF1 *expression level in the ECACC P0117 pedigree, whose affected individuals harbor an *NF1 *mutation predicted to result in exon 10a skipping (Figure 1). Presumably, this mutation does not affect mRNA stability. We observed similar mRNA transcript stability in the *Nf1*^+/- ^mouse (Figure 2) although the modest levels of *NF1 *expression in lymphocytes may preclude detection of expression differences. Similar transcript stability (as determined by northern blot analysis) was observed in the original publication describing the NF1 mouse model [15], although subsequent work using more sensitive techniques (quantitative PCR) have found modest expression differences in *Nf1 *in homogenized brain tissue from *Nf1*^+/-^*;Trp53*^+/-^*cis *mice [28].

We found few significant transcripts by permuted *t *test and intersection analysis shared among the Coriell-18, ECACC-6 and *Nf1*-Mouse-12 groups (Table 2, Figure 3 and Additional file 1), however few transcripts were validated by qPCR (Table 3). The small number of statistically significant transcripts may be due to the limited number of samples or high variability of individual gene expression levels in the samples. In summary, in LCLs *NF1*-haploinsufficiency appears to have a small effect on the expression of any single transcript.

Because we expect the changes in specific transcripts to be subtle between wild-type and *NF1*^+/- ^cells, we sought evidence for global changes in specific ontologic categories of genes (and not individual transcripts) perturbed by *NF1*-haploinsufficiency. We chose Gene Set Enrichment Analysis given its success in identifying *KRAS2 *expression [29], JNK signalling [24] and clinical survival signatures [30] in variety of datasets. To be conservative, we used 5000 permutations, set a low false discovery rate threshold (<0.05) and compared results across our three expression datasets. Using the leading edge analysis algorithm in GSEA, we observed striking similarities in the proportion and ontology of up- and down-regulated categories in the two human and one mouse group (Figure 5). Four of the five top up-regulated ontological categories in the ECACC-6 and *Nf1*-Mouse-12 groups were identical (cell cycle/mitosis/cytokinesis, DNA repair/replication/recombination, transcription/RNA processing, cell proliferation). Three of the four top up-regulated ontological categories in the Coriell-18 group (transcription/RNA processing, DNA repair/replication/recombination and cell proliferation) were among the top five categories in ECACC-6 and *Nf1*-Mouse-12 groups. The similarities between the ECACC-6 and *Nf1*-Mouse-12 groups may be due to similar mutation types: the *NF1 *mutation in both the ECACC-6 family and the *Nf1*^+/- ^mice results in in-frame exon skipping [15], whereas the mutation in the Coriell-18 group is predicted to lead to a premature stop codon. In the Coriell-18 group, the largest up-regulated category (translation/protein biosynthesis/ribosome biogenesis, accounting for ~40% of LEA genes) was not in the top 14 categories of the ECACC-6 and *Nf1*-Mouse-12 groups. Furthermore, there were fewer significant gene sets enriched in the Coriell-18 group (4 sets) than in the ECACC-6 (65 sets) and *Nf1*-Mouse-12 groups (189 sets), which may make meaningful statistical comparisons difficult.

The largest up-regulated category in the GSEA leading edge analysis of the ECACC-6 and *Nf1*-Mouse-12 groups is cell cycle/mitosis/cytokinesis (e.g. *CDC20*, *CDC2*, *FOXM1*, *MCM3*, *MCM6*, *MCM2*, *CCNB2 *- Additional file 4). This perturbation is consistent with the known RAS dysregulation observed in *NF1*-haploinsufficiency. The second-most perturbed up-regulated process in ECACC-6 and *Nf1*-Mouse-12 (and third-most perturbed in Coriell-18) groups is the DNA replication/repair/recombination category (e.g. *RFC4*, *FEN1*, *RFC3*, *UNG*, *RAD51 *- Additional file 4). This DNA damage response (DDR), or activation of genes associated with DNA replication/repair/recombination, is likely secondary to oncogene-associated (e.g. NF1-associated dysregulation of RAS) up-regulation of cell cycle/mitosis/cytokinesis genes [31]. Null or loss-of-function mutations in genes associated with DNA replication/repair/recombination are typically deleterious [32]. Paradoxically, activation of DDR genes themselves (especially in the context of on-going DNA replication stress) can result in oncogene-induced DNA damage, genomic instability and progression in human precancerous lesions [31]. To our knowledge, oncogene-induced DNA damage has not been specifically observed in NF1-associated tumors, although the murine *Nf1*^+/-^*;p53*^+/-^*cis *model of NF1 malignancies hosts a mild mutator phenotype in a wide variety of normal tissues in mice [33]. Our observations of DNA replication/repair/recombination up-regulation in germline *NF1*-haploinsufficiency suggest that subsequent *NF1 *bi-allelic inactivation and loss of heterozygosity may be secondary, in part, to oncogene-induced DNA damage. It is possible that the perturbations in cell cycle and DNA repair pathways that we observe apply to tumor suppressor haploinsufficiency in general, and not NF1 in particular. We are not aware of similar analyses in other tumor suppressor genes (e.g. *RB*).

These observations are largely supported by our analysis with Onto-Express, Pathway-Express and MetaCore tools (Biological Processes and Pathway Maps). These methods revealed perturbed cell cycle, mitosis, transcription and DNA replication and repair pathways, especially in up-regulated genes in the Coriell-18 and *Nf1*-Mouse-12 sets. Perturbed immune pathways were commonly identified in down-regulated genes in Coriell-18 and *Nf1*-Mouse-12 sets.

Haploinsufficiency (of any gene) is traditionally viewed as a passive state in which loss of one allele is insufficient to maintain the wild-type phenotype. Mouse modelling of *Nf1*-haploinsufficiency clearly shows a permissive tumorigenic environment in NF1, although the actual mechanism is unclear [4]. Evidence from other tumor-predisposition syndromes suggest that haploinsufficiency is an active state that facilitates cancer progression [3]. The perturbed, up-regulated pathways we observed, including those controlling DNA damage and repair, may functionally contribute to *NF1*-haploinsufficiency as an "active state" that ultimately promotes the loss of the wild-type allele [3].

## Methods

All studies were performed after review by appropriate NIH institutional review board and animal use and care committees.

### Culture of lymphoblastoid cell lines from human pedigrees

All NF1-affected and -unaffected banked lymphoblastoid cell lines (LCLs; Epstein-Barr virus (EBV)-transformed peripheral blood B lymphocytes) from two human kindreds with neurofibromatosis type 1 were obtained from the Coriell Institute (pedigree 2176; Camden, NJ, USA) and the European Cell and Culture Collection ("ECACC," pedigree P0117; Salisbury, Wiltshire, United Kingdom). Cultures were maintained in an incubator at 37°C with 5% CO_2 _in T25 flasks in medium with RPMI 1640 supplemented with 2 mM L-glutamine, 100 Units/mL penicillin, 100 μg/mL streptomycin and 15% heat inactivated fetal bovine serum (all tissue culture reagents were from Gibco/Invitogen, Carlsbad, CA, USA). Cultures were grown to a density from 0.9 × 10^6 ^to 1.3 × 10^6 ^cells/mL and harvested in Trizol reagent (Invitrogen, Carlsbad, CA, USA). To reduce batch effects, manipulation of NF1-affected and -unaffected cultures was randomized.

### *NF1 *genotyping of human pedigrees

The sample designated by the repository institution as the proband from each of the two kindreds was genotyped for mutations in the gene *NF1 *[34]. The presence or absence of the specific mutation was then verified by PCR and sequencing in all members of the kindred.

### Matched groups

We matched nine NF1-affected and nine NF1-unaffected adults (age >15 years) within Coriell pedigree 2176 ("Coriell-18") and three NF1-affected and three NF1-unaffected adults within ECACC pedigree P0117 ("ECACC-6") by age and gender (Table 1). The average age of NF1-affecteds and -unaffecteds in the Coriell-18 group was 37.9 ± 16.5 and 37.6 ± 12.5 years, respectively. (In the Coriell pedigree, two samples from adults without a specified age were included in both the affected and unaffected groups.) The average age of NF1-affecteds and -unaffecteds in the ECACC-6 group was 36.7 ± 3.5 and 35.3 ± 5.1 years, respectively.

### Evaluation of *Nf1*^+/- ^and wild-type mice

Six *Nf1*^+/- ^B6 [15] (three males, three females) and six wild-type (three males, three females) adult ("*Nf1*-Mouse-12") mice from 5 litters of the same line were euthanized at the age of eight months with CO_2 _and the weights of brain, heart, kidneys, and spleen were recorded. To rule out the confounding effects of an occult leukemia, glioma or pheochromocytoma, a complete blood count, lymph node sampling, spleen biopsy, sternum bone marrow, adrenal gland biopsy and whole-brain sections were collected and examined histologically. Males and females were harvested on two different days.

### Isolation of CD19+ spleen B lymphocytes from *Nf1*^+/- ^and wild-type mice

The dissected spleens were homogenized with the tip of a syringe plunger (3 mL syringe, slip tip, BD Biosciences, Discovery Labware, Bedford, MA, USA) and passed through a 70 μm nylon cell strainer (BD Biosciences) and purified using the CD19 mouse microbeads (Miltenyi Biotec, Auburn, CA, USA) on the autoMACS Separator (Miltenyi Biotec) using the possel_d program, following the manufacturer's directions. CD19+ and CD19- flow-through fractions were collected, and the purity was assessed by FACS on aliquots using GR1-FITC, B220-PE, CD3-PerCPCy5.5 and MAC1-APC antibodies (BD Pharmingen, San Diego, CA, USA). The remaining CD19+ and CD19- fractions were immediately lysed in 1 mL of the Trizol reagent (Invitrogen) and frozen at -80°C.

### RNA and DNA isolation

For RNA and DNA isolation, Trizol cell lysates were mixed with chloroform (1/5 of lysate volume), vortexed for one minute and centrifuged in a table-top centrifuge at 13,000 rpm for 15 min at 4°C. The aqueous phase was used for RNA isolation and the organic phase was used for DNA isolation. For total RNA isolation, the aqueous phase was mixed with equal volume of 70% ethanol and immediately loaded onto RNeasy mini columns (Qiagen, Valencia, CA, USA), with subsequent steps performed as per the manufacturer's protocol. The RNA quality was estimated on a 2100 Bioanalyzer, RNA 6000 Nano Chips (Agilent, Santa Clara, CA, USA). Samples with RNA integrity number (RIN) of 8.0 and above were used for further analysis. Total RNA from mouse spleen B lymphocytes was analyzed with the *Nf1 *TaqMan quantitative RT-PCR assay (Applied Biosystems, Foster City, CA, USA) per the manufacturer's instructions. Genomic DNA isolation from Trizol lysates was performed as previously reported [35].

### Protein isolation and western blotting

Whole cell lysates for protein analysis were prepared using RIPA lysis buffer (Upstate Biotechnology (Millipore), Billerica, MA, USA) as per the manufacturer's instructions. Western blot analysis was done as described elsewhere [36,37]. All antibodies were obtained from Santa Cruz Biotechnology (Santa Cruz, CA, USA) and used as per the manufacturer's instructions. Signals were developed with the Super Signal West Pico chemiluminescent substrate (Pierce Biotechnology Inc., Rockford, IL, USA) and visualized by using Amersham Hyperfilm ECL X-ray film (GE Healthcare, Piscataway, NJ, USA). Western blots were stripped for hybridization with other primary antibodies in Restore Western Blot Stripping Buffer (Pierce) as specified in the manufacturer's protocol.

### Microarray expression profiling of LCLs and murine spleen CD19+ B lymphocytes

We performed microarray expression profiling of the human and mouse samples on single-color Illumina microarrrays. The human Coriell-18 group was hybridized to the Illumina platform as well as a two-color oligonucleotide array. Illumina expression datasets were submitted to Gene Expression Omnibus (GSE18444 - Coriell-18; GSE18445 - ECACC-6; GSE18447 - *Nf1*-Mouse-12; GSE18448 - all sets; two-color oligonucleotide microarray dataset is available upon request).

#### Illumina (single-color) microarray gene expression profiling

All reagents, consumables, lab-ware, instruments, and software were obtained from Illumina Inc., (San Diego, CA, USA) unless indicated otherwise. RNA amplification/labeling, microarray hybridization, and microarray washing/staining and scanning procedures were done according to the Illumina protocols without modifications. Amplified biotinylated cRNA (1.5 μg) was hybridized to either HumanWG-6_v2 or MouseWG-6_v1 Sentrix BeadChips. In all steps, care was exercised to avoid batch effects. Samples were hybridized to the microarrays at 55°C for 16-17 hours. Microarrays were washed to remove non-specifically bound cRNA, stained with 1 μg/mL Streptavidin-Cy3 (GE Healthcare, Piscataway, NJ, USA), dried, and scanned in BeadStation 500 scanner. Image acquisition and initial image analysis were done with Illumina BeadScan and BeadStudio applications.

#### Spotted oligonucleotide (two-color) microarray gene expression profiling

Microarray slide preparation, probe labeling and hybridization and data analysis were described elsewhere with some modifications [38,39]. Briefly, microarray slides were manufactured at the NHGRI microarray core facility from 34,580 oligonucleotide probes obtained from the Human Genome Oligonucleotide Set Version 3.0 from Qiagen Inc. (Valencia, CA, USA). Dried oligonucleotides were resuspended in 3× SSC solution and spotted on epoxy-coated slides. The slides were then cleaned by vigorous shaking in a 0.5% SDS solution for 2 min and incubated for 20 min in water at 50°C. The slides were dried by centrifugation. Total RNA (15 μg) from LCL samples or 15 μg of Universal Human Reference RNA (Stratagene/Agilent Technologies, La Jolla, CA, USA) were reverse transcribed with anchored oligo-dT primer in the presence of an aminoallyl dUTP. After purification, the cDNAs from LCLs were coupled with Cy3 and Universal Human Reference RNA - with Cy5 for 1 h. Labelled samples were column-purified from unincorporated Cy3 and Cy5. For each sample, total dye incorporation and total amount of synthesized cDNA were calculated. Samples with incorporated dye of greater than 30 picomoles per sample and a nucleotide/dye ratio of less than 50 were used for the hybridization step. The labelled cDNAs were then hybridized to the slides in a 1× In Situ Hybridization buffer (Agilent Technologies, Palo Alto, CA, USA). The hybridization was done overnight at 60°C in a rotisserie oven (Agilent Technologies). All subsequent steps were done in an ozone-free chamber to prevent Cy5 degradation. Slides were washed in a series of SSC/SDS buffers and dried by centrifugation. Scanning of microarrrays was done with a laser confocal scanner (Agilent Technologies), and the fluorescence intensities were measured in the spots and their surrounding areas.

### Statistical analysis of microarray data

#### Permuted t test and multiple testing correction

Raw data from the Illumina BeadChips for both human and mouse experiments was corrected for background and quantile normalized using BeadExplorer (version1.5.0), a Bioconductor module developed for quality control, normalization, annotation and exploration of Illumina BeadChip data [40]. Raw data from arrays from the NHGRI Microarray Facility was corrected for background and normalized by median shift using the DEARRAY IPLab image processing package (Scanalytics, Fairfax, VA, USA). All expression values were log_2 _transformed. Expression datasets were formatted and imported into BRB-ArrayTools (developed by Drs. Richard Simon and Amy Peng Lam, version 3.4.1) [41] and ArrayAnalysis [42] statistical packages. To identify statistically significant genes, we used the "Class comparison" and "F-test/T-test" analyses in BRB-ArrayTools and ArrayAnalysis, respectively. The false discovery rate (FDR), a permutation-based approach for multiple comparisons problem was used for identification of statistically significant genes.

#### Intersection (hypergeometric) analysis: Coriell-18 vs ECACC-6 vs Nf1-Mouse-12

Transcripts in the two human and mouse groups were ranked by their nominal *P *values and the top ~5% of genes from each list were chosen for intersection analysis. Before comparison, all mouse transcripts were converted to their human homologues. We used the hypergeometric distribution function and Fisher exact test to determine the significance of the overlap of specific genes between the human and mouse groups.

#### Intersection (hypergeometric) analysis: Illumina vs. spotted oligonucleotide arrays

Permuted *t *tests were performed for the expression dataset on Coriell-18 from both the Illumina and spotted oligonucleotide arrays. After ranking transcripts by their nominal *P *values and filtering out transcripts not expressed in both expression datasets, the significance of the intersection analysis for the ~5% top-ranking genes from the lists was determined using the hypergeometric distribution function and Fisher exact test.

### Quantitative real-time reverse transcriptase PCR

We selectively validated a group of 22 transcripts (twelve human and ten mouse) for quantitative PCR (qPCR) validation. We chose transcripts that were either statistically significant by *t *tests or intersection analysis. All reagents, consumables, instruments, protocols, and software used in the gene expression analyses were obtained from Applied Biosystems. Total RNA samples were adjusted to 100 ng/μL and used for cDNA synthesis with High Capacity cDNA Archive Kit according to the manufacturer's protocol. The cDNA preps were used in real-time qPCR without further purification and were kept frozen at - 20°C when not in use. All real-time qPCR analyses in this study were performed with TaqMan gene expression assays. One μL of an unpurified cDNA mix was used as an input for an individual qPCR reaction. TaqMan Universal PCR Master Mix, cDNA and TaqMan gene expression assays were mixed according to the manufacturer's protocol. Reactions were performed in 384-well plates with 20 μL of reaction mix per well. Expression analysis of each gene in each sample was done in triplicates. Human and mouse *GAPDH/Gapdh *gene expression assays were used as normalization controls. All reactions were done in a 7900HT Fast Real-Time PCR System instrument using the default amplification protocol. Relative quantitation of gene expression was done using "double delta Ct" method according to the manufacturer's protocol.

### Gene Set Enrichment Analysis (GSEA)

The GSEA analysis was performed as described elsewhere [43]. Briefly, Illumina expression datasets were background-corrected and quintile normalized and loaded into the application along with phenotype label and chip annotations files. Analysis was performed against the entire GSEA database (June 2009), which after filtering out gene sets smaller than 15 and larger than 500 genes (as recommended) included ~3400 gene sets. "Permutation type" parameter was chosen as "gene-set" for all sample sets, since one of them (ECACC-6) was comprised of fewer than twelve samples. Five thousand permutations were performed for each sample set. The FDR threshold for statistically significant gene sets was set at 0.05. Gene sets with FDR at or below 0.05 were submitted to a leading edge analysis (LEA). LEA extracts the genes that contributed most significantly to the enrichment score. An ontological analysis ("Biological Process" in Gene Ontology and UniProt databases) of the LEA gene lists was performed for the genes, which were present in at least 10% of statistically significant gene sets submitted for the LEA. In cases when fewer than 20 significant gene sets were identified, we either performed LEA with gene sets with the best 20 FDR scores (*Nf1*-Mouse-12 down-regulated gene sets) or performed ontological analysis for the genes in the LEA list which were present in at least two gene groups (Coriell-18 up-regulated gene sets). For ontological analysis, each gene was placed into one or more categories, and closely related categories were bundled together (for instance, "DNA repair", "DNA replication", "DNA damage", "DNA recombination" categories were bundled into the "DNA repair/replication/recombination" category). There was a single significant gene set down-regulated in ECACC-6 group, and therefore the LEA could not be performed.

### Ontological analysis of differentially expressed genes with Onto-Express, Pathway-Express and MetaCore tools

Up- and down-regulated differentially expressed genes (*t *test *P *< 0.05) in Coriell-18, ECACC-6 and *Nf1*-Mouse-12 sample sets were subject to ontological analysis with Onto-Express [16,17], Pathway-Express [18,19] and MetaCore tools (GeneGo, Inc.) as per the authors' or manufacturer's guidelines. In the Onto-Express and MetaCore analyses, a hypergeometric distribution was used for calculating nominal *P *values and the FDR procedure was used for correction of multiple testing. In the Pathway-Express analysis, the default settings were used except for the "Correction" option; FDR was selected as a multiple testing correction procedure. Ontological categories and pathways with FDR values equal or less than 0.05 were considered significant.

## Abbreviations

NF1: neurofibromatosis type 1; LCL: lymphoblastoid cell line; GSEA: gene set enrichment analysis; EBV: Epstein-Barr virus; FACS: fluorescent activated cell sorting; FDR: false discovery rate; LEA: leading edge analysis; DDR: DNA damage response; ECACC: the European cell and culture collection.

## Authors' contributions

AP carried out collection, analysis and interpretation of data and drafted the manuscript. CP assisted with collection and analysis of data. KMR provided mice and assisted with interpretation of data. DRS conceived and designed the study, interpreted data and wrote the final draft of the manuscript. All authors read and approved the final manuscript.

## Supplementary Material

Additional file 1 **Intersection analysis of Coriell-18, ECACC-6 and *Nf1 *-Mouse-12 expression datasets**. The most differentially expressed genes (top 5 per cent) from the human and mouse sets were used for intersection analysis. The results of a pair-wise and three-way intersection analysis are shown. See also Figure 3. Click here for file

Additional file 2 **Comparison of fold difference values of the most differentially expressed genes on Illumina and NHGRI microarrays**. Samples from Coriell-18 set were analyzed in parallel on Illumina and NHGRI microarrays. Intersection analysis of the most differentially expressed transcripts from two platforms was performed, and the expression fold difference (Affecteds vs. Unaffecteds) was calculated for each transcript found in the overlap between the two platforms. See also Figure 4. Click here for file

Additional file 3 **Quantitative PCR validation of microarray data for select human and mouse genes**. Twelve human genes were subject to qPCR validation in the human Coriell-18 and ECACC-6 sets. Ten mouse genes were subject to qPCR validation in the murine *Nf1*-Mouse-12 set. Both microarray and qPCR expression values in NF1-affecteds and *Nf1*^+/- ^mice were normalized to expression values in NF1-unaffecteds or wild-type mice, respectively. Expression values in NF1-unaffecteds and wild-type mice were arbitrary set at 1.0. Red bars denote mean expression in NF1-affecteds on microarrays, blue bars denote mean expression in NF1-unaffecteds on microarrays; orange bars denote mean expression in NF1-affecteds by qPCR; and green bars denote mean expression in NF1-unaffecteds by qPCR. Gene names are shown below each set of bars. Sample set names are shown on top of each plot. Error bars are equal to one standard deviation. Asterisks above bars denote genes validated by qPCR (nominal *P *value < 0.05). Click here for file

Additional file 4 **Leading edge analysis identifies transcripts that are most frequently present in the significant gene sets**. Leading edge analysis of significant GSEA gene sets was performed, and the transcripts were ranked by the total number of times they are present in statistically significant gene sets. See also Figure 5. Click here for file

Additional file 5 **Ontological annotation of transcripts identified by leading edge analysis**. Up- and down-regulated transcripts identified by leading edge analysis were ontologically annotated, and similar or related categories were combined. The resulting categories were ranked by the number of transcripts included in each category. Click here for file

Additional file 6 **Ontological analysis of differentially expressed genes with Onto-Express**. Functional profiles based on Gene Ontology (GO) biological processes terms were created for up- and down-regulated transcripts in two human and a mouse sample sets. For each set of transcripts, statistically significant ontological categories were determined by the hypergeometric test, followed by a multiple testing correction procedure (FDR). Only categories with FDR below the threshold (0.05) are shown. Note that there are no significant categories in the lists of down-regulated transcripts in ECACC-6 and *Nf1*-Mouse-12 sets. Click here for file

Additional file 7 **Pathway level analysis of differentially expressed genes with Pathway-Express**. Differentially expressed genes in two human and a mouse sample sets were compared to known cellular pathways in the KEGG database, followed by impact factor computation and FDR correction. Statistically significant pathways (FDR < 0.05) and their impact factors (IF) are shown for each sample set. Click here for file

Additional file 8 **Ontological analysis of differentially expressed genes with MetaCore tools**. Ontological profiling similar to that described in Additional file 7 was performed using proprietary MetaCore software and GO database. *P *values and FDR (not shown in the tables) were calculated for each category. Only categories with FDR below threshold (0.05) are shown. Note that there are no significant categories in the set of *Nf1*-Mouse-12 down-regulated transcripts. Click here for file

Additional file 9 **Pathway level analysis of differentially expressed genes with MetaCore tools**. Up- and down-regulated genes in two human and a mouse sample sets were analyzed with GeneGo pathway maps database. *P *values and FDR (not shown in the tables) were calculated for each pathway. Only pathways with FDR below threshold (0.05) are shown. Note that there are no significant pathways in the sets of down-regulated transcripts. Click here for file

Additional file 10 **Dynamic change of neurofibromin level in lymphoblastoid cell lines in response to serum deprivation**. We determined the effects of serum deprivation on neurofibromin level as a way to establish the physiologic relevance of lymphoblastoid cell lines (LCLs) in the study of *NF1*-haploinsufficiency. We measured levels of neurofibromin in two LCLs (one each from NF1-affected, and -unaffected individuals) that were serum-deprived (0.1% serum) for 16 hours, and then released for variable amounts of time in complete (10% serum; supports cell proliferation), or incomplete (1% serum; does not support cell proliferation) medium. Western blot analysis and quantitation of relative abundance of neurofibromin in an NF1-unaffected individual (A) and an NF1-affected individual (B). NF1 abundance data is shown to the right from respective western blot and is plotted as percentage relative to *NF1 *abundance in exponentially growing cells. "E" - exponentially growing LCLs; "S" - serum starved LCLs; 5', 30', 7 h - cells released into media containing either 1% or 10% FBS for 5 min, 30 min or 7 hours, respectively. Our experiment showed that the amount of neurofibromin increased approximately two-fold in serum-starved cells as compared to that in exponentially growing LCLs from both affected and unaffected individuals. When the cells were released into complete medium (10% FBS), the neurofibromin level quickly returned to pre-starvation levels in both NF1-affected and -unaffected LCLs. In contrast, the neurofibromin level continued to increase during prolonged incubation of the cells in incomplete medium (1% FBS). We conclude that in LCLs neurofibromin level is sensitive to environmental conditions. Click here for file
