# Supplementary figures and images for: Evidence of perturbations of cell cycle and DNA repair pathways as a consequence of human and murine NF1-haploinsufficiency
Source: BMC Genomics. 2010 Mar 22;11:194. doi: 10.1186/1471-2164-11-194 (PMC2858150; doi:10.1186/1471-2164-11-194)

# Relative Gene Expression

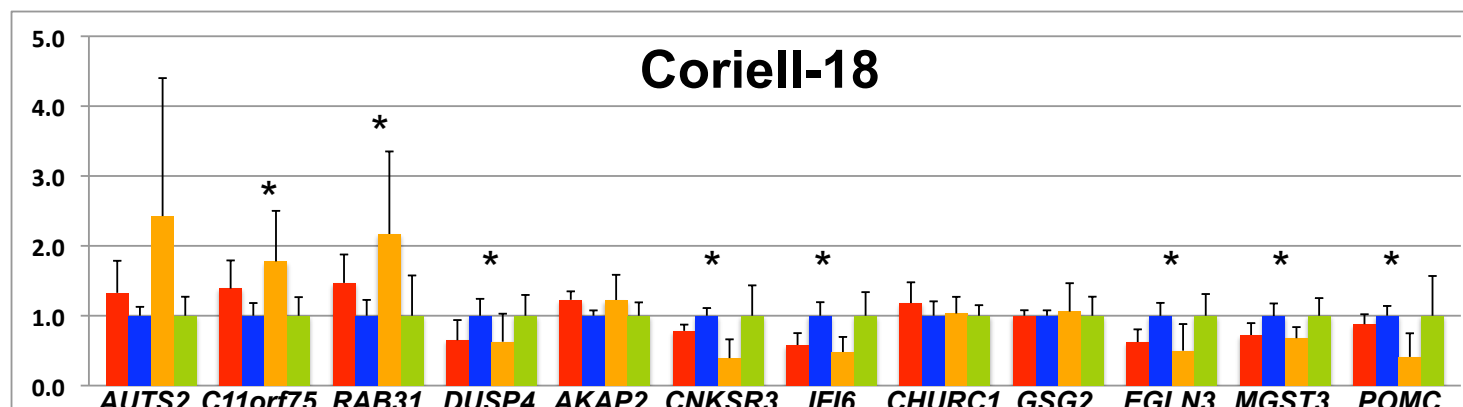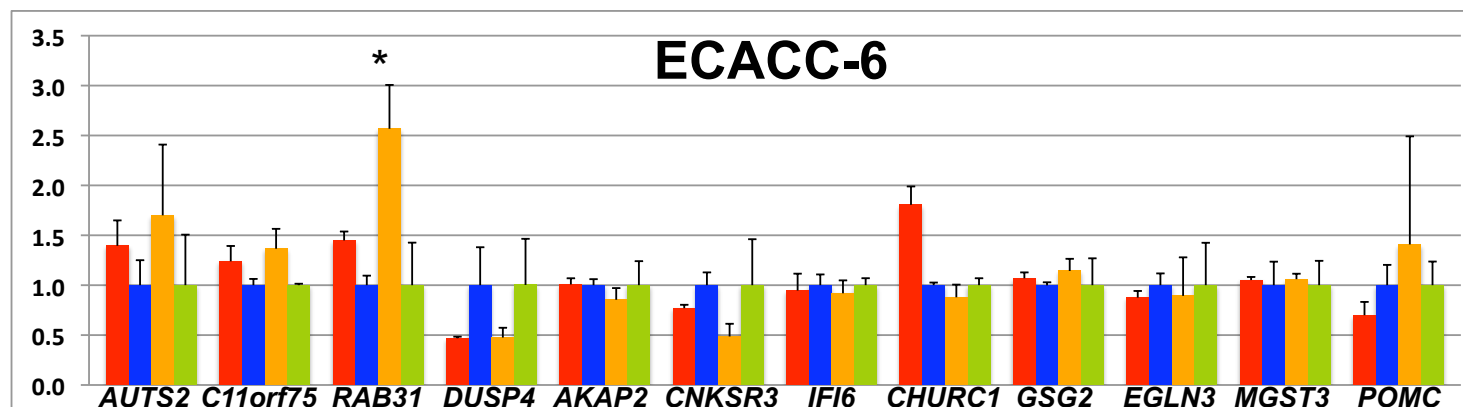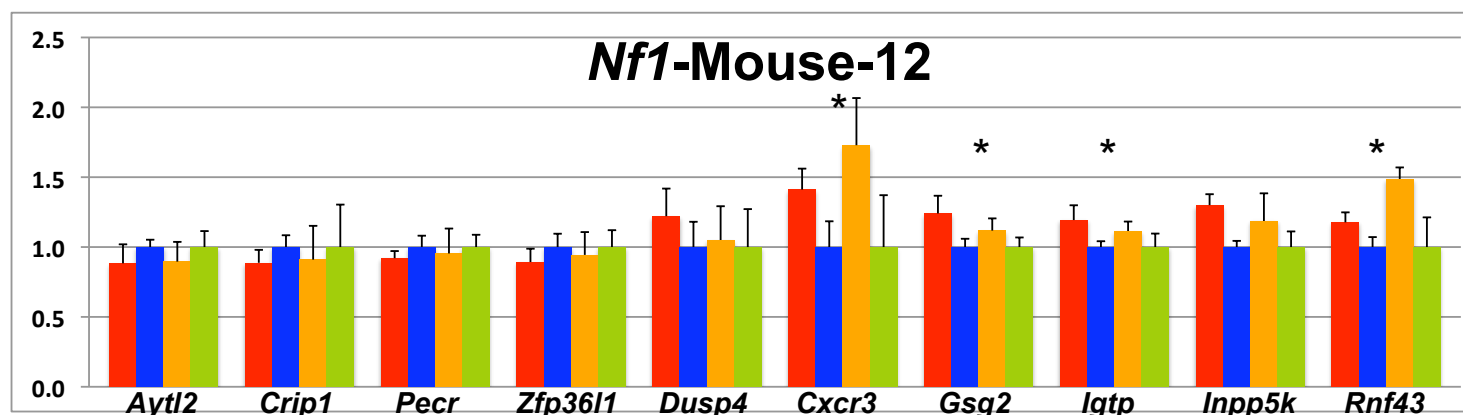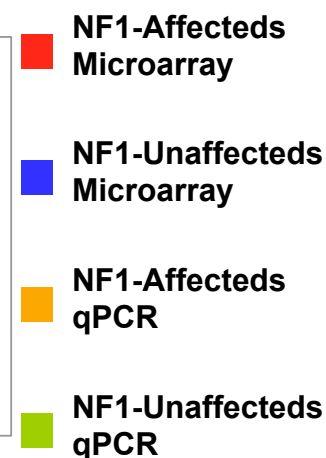

Supplement: Additional file 3 — Quantitative PCR validation of microarray data for select human and mouse genes. Twelve human genes were subject to qPCR validation in the human Coriell-18 and ECACC-6 sets. Ten mouse genes were subject to qPCR validation in the murine Nf1-Mouse-12 set. Both microarray and qPCR expression values in NF1-affecteds and Nf1+/- mice were normalized to expression values in NF1-unaffecteds or wild-type mice, respectively. Expression values in NF1-unaffecteds and wild-type mice were arbitrary set at 1.0. Red bars denote mean expression in NF1-affecteds on microarrays, blue bars denote mean expression in NF1-unaffecteds on microarrays; orange bars denote mean expression in NF1-affecteds by qPCR; and green bars denote mean expression in NF1-unaffecteds by qPCR. Gene names are shown below each set of bars. Sample set names are shown on top of each plot. Error bars are equal to one standard deviation. Asterisks above bars denote genes validated by qPCR (nominal P value < 0.05). [file 1471-2164-11-194-S3.PDF]

## A NF1-Unaffected

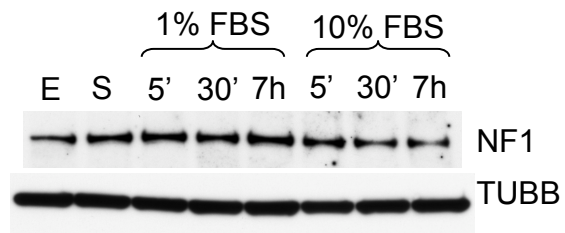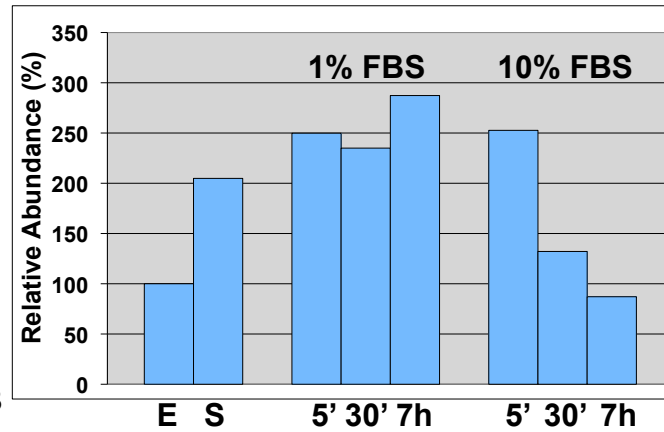

## B NF1-Affected

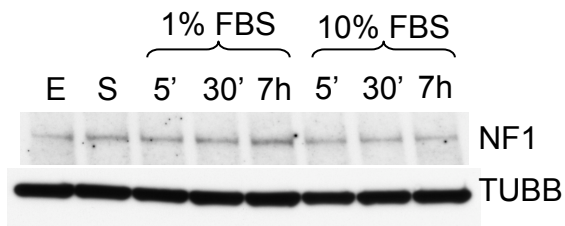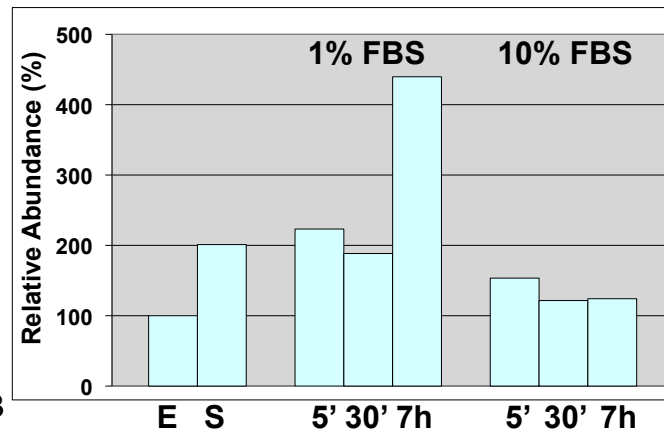

Supplement: Additional file 10 — Dynamic change of neurofibromin level in lymphoblastoid cell lines in response to serum deprivation. We determined the effects of serum deprivation on neurofibromin level as a way to establish the physiologic relevance of lymphoblastoid cell lines (LCLs) in the study of NF1-haploinsufficiency. We measured levels of neurofibromin in two LCLs (one each from NF1-affected, and -unaffected individuals) that were serum-deprived (0.1% serum) for 16 hours, and then released for variable amounts of time in complete (10% serum; supports cell proliferation), or incomplete (1% serum; does not support cell proliferation) medium. Western blot analysis and quantitation of relative abundance of neurofibromin in an NF1-unaffected individual (A) and an NF1-affected individual (B). NF1 abundance data is shown to the right from respective western blot and is plotted as percentage relative to NF1 abundance in exponentially growing cells. "E" - exponentially growing LCLs; "S" - serum starved LCLs; 5', 30', 7 h - cells released into media containing either 1% or 10% FBS for 5 min, 30 min or 7 hours, respectively. Our experiment showed that the amount of neurofibromin increased approximately two-fold in serum-starved cells as compared to that in exponentially growing LCLs from both affected and unaffected individuals. When the cells were released into complete medium (10% FBS), the neurofibromin level quickly returned to pre-starvation levels in both NF1-affected and -unaffected LCLs. In contrast, the neurofibromin level continued to increase during prolonged incubation of the cells in incomplete medium (1% FBS). We conclude that in LCLs neurofibromin level is sensitive to environmental conditions. [file 1471-2164-11-194-S10.PDF]
